# Effects of puerarin on the pharmacokinetics of triptolide in rats

**DOI:** 10.1080/13880209.2019.1626448

**Published:** 2019-06-22

**Authors:** Qingfa Wang, Yanping Wu, Fengting Xiang, Yan Feng, Zhenghao Li, Yufeng Ding

**Affiliations:** a Department of Neonatology, Yidu Central Hospital of Weifang, Shandong, China;; b Department of Pharmacy, Yidu Central Hospital of Weifang, Shandong, China

**Keywords:** Caco-2 cells, *P-gp*, metabolism

## Abstract

**Context:** Puerarin and triptolide are sometimes used together for the treatment of disease in Chinese clinics; however, the drug–drug interaction between puerarin and triptolide is still unknown.

**Objective:** This study investigates the effects of puerarin on the pharmacokinetics of triptolide in rats and clarifies its main mechanism.

**Materials and methods:** The pharmacokinetic profiles of oral administration of triptolide (1 mg/kg) in Sprague-Dawley rats with (test group, *n* = 6) or without pretreatment (control group, *n* = 6) with puerarin (100 mg/kg/day for seven days) were investigated. The effects of puerarin on the transport and metabolic stability of triptolide were also investigated using Caco-2 cell transwell model and rat liver microsomes.

**Results:** The results showed that puerarin could significantly increase the peak plasma concentration (from 187.25 ± 15.36 to 219.67 ± 21.52 ng/mL), and decrease its oral clearance (from 4.92 ± 0.35 to 62.46 ± 3.75 ± 0.19 L/h/kg). The Caco-2 cell transwell experiments indicated that puerarin could decrease the efflux ratio of triptolide from 2.70 to 1.33, and the intrinsic clearance rate of triptolide was decreased by the pretreatment with puerarin (38.8 ± 4.7 vs. 32.9 ± 6.5 μL/min/mg protein).

**Discussion and conclusions:** Puerarin could significantly change the pharmacokinetic profiles of triptolide in rats, and it might exert these effects through increasing the absorption of triptolide by inhibiting the activity of *P-gp*, or through inhibiting the metabolism of triptolide in rat liver. The results also showed that the dose of triptolide should be decreased when these drugs were co-administered.

## Introduction

Triptolide, a diterpenoid triepoxide, is a major pharmacological component isolated from *Tripterygium wilfordii* Hook F (Celastraceae) (Brinker and Raskin 2005; Jin et al. 2009; Su et al. 2014), and it has been used primarily for the treatment of inflammatory and autoimmune diseases such as rheumatoid arthritis, systemic lupus erythematosus, skin diseases and cancer (Liu et al. 2011a, 2011b; Cheng et al. 2014; Hu et al. 2014; Li et al. 2014; Ling et al. 2014; Wang et al. 2014; Wei and Huang 2014). However, the clinical application of triptolide was restricted because of its narrow therapeutic range and severe toxicity to digestive, reproductive and hematopoietic systems (Li et al. 2014; Singla and Challana 2014).

Puerarin is an active and main constituent isolated from Pueraria Radix, the root of *Pueraria lobata* (Willd) Ohwi (Fabaceae) (Yeung et al. 2006). Puerarin is widely used in China for the treatment of cardiovascular diseases and diabetes (Wong et al. 2011). Several studies have also indicated that puerarin possesses antioxidant, antiplatelet, antiinflammatory, antiarrhythmic and antiapoptotic properties (Choo et al. 2002; Liu et al. 2011a, 2011b; Zhang et al. 2011; Huang et al. 2012). Previous studies have reported that puerarin could inhibit the activity of CYP3A4 and *P-gp* (Guo et al. 2014; Kim et al. 2014), which might lead to drug–drug interactions when they are co-administered with other herbs or drugs that are *P-gp* substrates. Puerarin are always used together with other herbs or drugs in Chinese traditional medicines, most studies investigated the effects of other herbs or drugs on the pharmacokinetics of puerarin (Liao et al. 2014; Liu et al. 2018; Zhao et al. 2018; Zhou et al. 2018; Zhang et al. 2019). However, few studies have investigated the effects of puerarin on the pharmacokinetics of other co-administered drugs or herbs. Puerarin and triptolide are sometimes used together for the treatment of disease in clinics in China. However, the drug–drug interaction between puerarin and triptolide is still unknown.

This study investigates the effects of puerarin on the pharmacokinetics of triptolide in rats. First, the *in vivo* pharmacokinetics of triptolide in rats with or without pretreatment with puerarin was investigated. Then, the effects of puerarin on the transport of triptolide were investigated using the Caco-2 cell transwell model, and the effects of puerarin on the metabolic stability of triptolide were studied using rat liver microsomes.

## Materials and methods

### Chemicals and reagents

**Figure 1.**
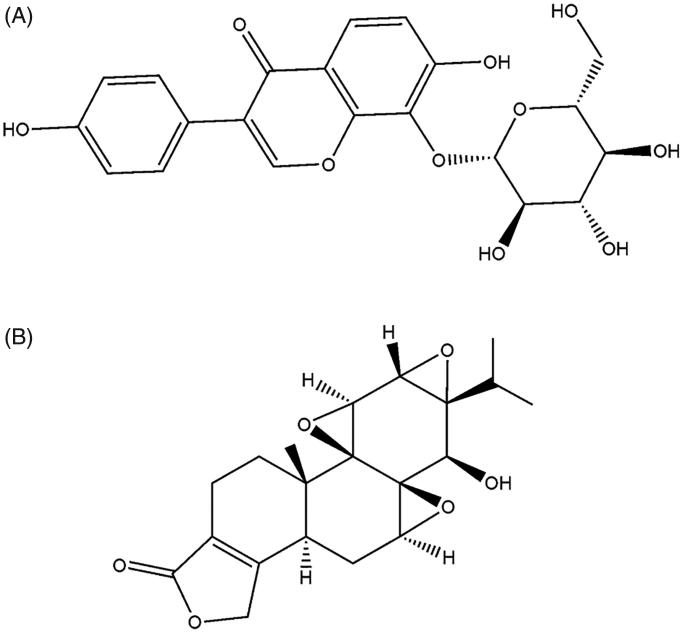
The chemical structures of puerarin (A) and triptolide (B).

Triptolide (purity > 98%) and puerarin (purity > 98%) were purchased from the National Institute for the Control of Pharmaceutical and Biological Products (Beijing, China), and the structures are shown in Figure 1. Rat liver microsomes were purchased from BD Gentest™ (Becton Dickinson, Franklin Lakes, NJ, USA). Dulbecco’s modified Eagle’s medium (DMEM) was purchased from Thermo Scientific Corp. (Logan, UT, USA). Hanks' balanced salt solution (HBSS) was purchased from GIBCO (Grand Island, NY, USA). Acetonitrile and methanol were purchased from Fisher Scientific (Fair Lawn, NJ, USA). Formic acid was purchased from Anaqua Chemicals Supply Inc. Limited (Houston, TX, USA). Ultrapure water was prepared with a Milli-Q water purification system (Millipore, Billerica, MA, USA). All other chemicals were of analytical grade or better.

### Animal experiments

Male Sprague-Dawley (SD) rats weighing 220–250 g were provided by the Experimental Animal Center of the Weifang Medical University (Weifang, China). Rats were bred in a breeding room at 25 °C with 60 ± 5% humidity and a 12 h dark–light cycle. Tap water and normal chow were given *ad libitum*. All of the experimental animals were housed under the above conditions for a three-day acclimation period and fasted overnight before the experiments. The animal facilities and protocols were approved by the Institutional Animal Care and Use Committee (No. 12, 2018, Ethical Research of Weifang Medical University). All procedures were performed in accordance with the National Institutes of Health guidelines regarding the principles of animal care.

### *In vivo* pharmacokinetic study

To evaluate the effects of puerarin on the pharmacokinetics of triptolide, the rats were divided into two groups of six animals each. The test group was orally pretreated with puerarin at a dose of 100 mg/kg/day (dissolved directly in normal saline containing 0.5% methylcellulose at a concentration of 2 mg/mL) for seven days before the administration of triptolide (Zhao et al. 2018). Next, triptolide were orally administered to rats by gavage at a dose of 1 mg/kg (Wang et al. 2017). Blood samples (250 μL) were collected into heparinized tubes via the *oculi chorioideae* vein at 0, 2, 5, 10, 15, 30, 45, 60, 90, 120, 180, 240 and 360 min after the oral administration of triptolide. The blood samples were centrifuged at 3500 rpm for 5 min. The plasma samples that were obtained were stored at –40 °C until analysis.

### Instruments and conditions

The LC–MS/MS method was performed according to the previous report by Zhang et al. (2016). The analysis was performed on an Agilent 1290 series liquid chromatography system (Agilent Technologies, Palo Alto, CA, USA), including a binary pump, an on-line vacuum degasser, a surveyor autosampling system, a column temperature controller, and an Agilent 6460 triple-quadruple mass spectrometer (Agilent Technologies, Palo Alto, CA, USA) with Turbo Ion spray, which is connected to the liquid chromatography system. An Agilent MassHunter B 4.0 software was used for the control of equipment, data acquisition, and Agilent Quantitative analysis software was used for data analysis. The chromatographic analysis of triptolide was performed on a Shiseido MG-C18 column (3.0 × 100 mm, i.d.; 3.0 μm, Tokyo, Japan) at room temperature. The mobile phase was water (containing 0.1% formic acid) and acetonitrile (30:70, v:v) at a flow rate of 0.6 mL/min, and the split ratio was 1:1.

The mass scan mode was positive MRM mode. The precursor ion and product ion are *m/z* 361.3 → 128.2 for triptolide, and *m/z* 363.5 → 121.0 for IS, respectively. The collision energy for triptolide and IS was 30 and 20 eV, respectively. The MS/MS conditions were optimized as follows: fragmentor, 110 V; capillary voltage, 3.5 kV; nozzle voltage, 500 V; nebulizer gas pressure (N_2_), 40 psig; drying gas flow (N_2_), 10 L/min; gas temperature, 350 °C; sheath gas temperature, 400 °C; sheath gas flow, 11 L/min.

### Data analysis

The pharmacokinetic parameters, including the area under the plasma concentration–time curve (AUC), maximal plasma concentration (*C*_max_), the time for the maximal plasma concentration (*T*_max_) and the mean residence time (MRT) were calculated using the DAS 3.0 pharmacokinetic software (Chinese Pharmacological Association, Anhui, China).

The differences between the mean values were analysed for significance using a one-way analysis of variance (ANOVA). Values of *p <* 0.05 were considered to be statistically significant.

### Cell culture

The Caco-2 cell line was obtained from the American Type Culture Collection (Manassas, VA). The Caco-2 cells were cultured in DMEM high glucose medium containing 15% FBS, 1% NEAA and 100 U/mL penicillin and streptomycin. The cells were cultured at 37 °C with 5% CO_2_. For transport studies, the cells at passage 40 were seeded on transwell polycarbonate insert filters (1.12 cm^2^ surface, 0.4 μm pore size, 12 mm diameter; Corning Costar Corporation, Cambridge, MA) in 12-well plates at a density of 1 × 10^5^ cells/cm^2^. Cells were allowed to grow for 21 days. For the first seven days, the medium was replaced every two days, and then daily. The transepithelial electrical resistance (TEER) of the monolayer cells was measured using Millicell ERS-2 (Millipore Corporation, Billerica, MA), and TEER exceeding 400 Ω/cm^2^ was used for the flux experiment. The integrity of the Caco-2 monolayers was confirmed by the paracellular flux of Lucifer yellow, which was less than 1% per hour. The alkaline phosphatase activity was validated using an Alkaline Phosphatase Assay Kit. The qualified monolayers were used for transport studies.

### Effects of puerarin on the absorption of triptolide in the Caco-2 cell transwell model

Before the transport experiments, the cell monolayers were rinsed twice using warm (37 °C) HBSS, then the cells were incubated at 37 °C for 20 min. After preincubation, the cell monolayers were incubated with triptolide in fresh incubation medium added on either the apical (AP) or basolateral (BL) side for the indicated times at 37 °C. The volume of incubation medium on the AP and BL sides was 0.5 mL and 1.5 mL, respectively, and a 100 μL aliquot of the incubation solution was withdrawn at the indicated time points from the receiver compartment and replaced with the same volume of fresh pre-warmed HBSS buffer. The inhibitory effects of *P-gp* inhibitors on the triptolide flux by Caco-2 cells were investigated by adding 50 μM puerarin to both sides of the cell monolayers and preincubating the sample at 37 °C for 30 min. The permeability of triptolide (2 μM) in all of the above conditions for both directions, i.e., from the AP side to the BL side and from the BL side to the AP side, was measured after incubation for 30, 60, 90 and 120 min at 37 °C. In addition, the efflux activity of *P-gp* was validated using a typical *P-gp* substrate, digoxin (25 μM).

The apparent permeability coefficient (*P*_app_) was calculated using the equation of Artursson and Karlsson:

$$P_{\text{app}}=\left(\text{Δ}Q/\text{Δ}t\right)\times \left[1/\left(A\times C_{0}\right)\right]$$

where *P*_app_ is the apparent permeability coefficient (cm/s), Δ*Q*/Δ*t* (μmol/s) is the rate at which the compound appears in the receiver chamber, *C*_0_ (μmol/L) is the initial concentration of the compound in the donor chamber and *A* (cm^2^) represents the surface area of the cell monolayer. Data were collected from three separate experiments, and each was performed in triplicate.

### Effects of puerarin on the metabolic stability of triptolide in rat liver microsomes

Rat liver microsomes were used to investigate the effects of puerarin on the metabolism clearance of triptolide, and the assay conditions and reaction mixtures were similar to those reported previously (Wang et al. 2017; Yan et al. 2017). In brief, 30 μL rat liver microsome (20 mg/mL), 12 μL triptolide solution (100 μM) and 1113 μL PBS buffer (0.1 M, pH 7.4) were added to the centrifuge tubes on ice. There was a 5 min preincubation step at 37 °C before initiating the reaction by adding NADPH-generating system (45 μL) into the microsomal suspension. The effects of puerarin or ketoconazole (a positive CYP3A4 inhibitor) on the metabolic stability of triptolide were investigated by adding 10 μM of puerarin or ketoconazole (12 μL, final concentration of 0.1 μM) to rat liver microsomes and preincubating them for 30 min at 37 °C, followed by the addition of NADPH-generating system. Aliquots of 100 μL were collected from the reaction volumes at 0, 1, 3, 5, 15, 30 and 60 min after the addition of triptolide, and 200 μL ice-cold acetonitrile containing esculin was added to terminate the reaction. All the experiments were performed in triplicate. The subsequent sample preparation method was the same as the plasma sample preparation method, and the concentration of triptolide was determined by LC–MS.

The *in vitro* half-life (*t*_1/2_) was obtained using the equation: *t*_1/2_ = 0.693/*k*; *V* (μL/mg) = volume of incubation (μL)/protein in the incubation (mg); intrinsic clearance (Clint) (μL/min/mg protein) = *V* × 0.693/*t*_1/2_.

## Results

### Effects of puerarin on the pharmacokinetics of triptolide

**Figure 2.**
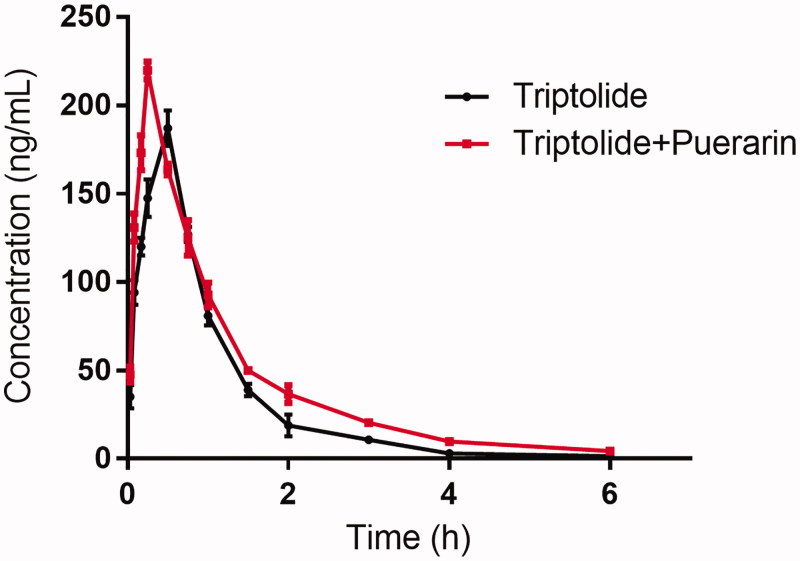
The pharmacokinetic profiles of triptolide in rats after the oral administration of 1 mg/kg triptolide with or without puerarin pretreatment (100 mg/kg/day for seven days).

The mean plasma concentration–time curves of oral administration of triptolide in male SD rats with or without pretreatment with puerarin are presented in Figure 2.

The pharmacokinetic parameters of triptolide were calculated using the noncompartmental method with the DAS 3.0 pharmacokinetic software (Chinese Pharmacological Association, Anhui, China). The pharmacokinetic parameters are shown in Table 1.

**Table 1.** Pharmacokinetic parameters of triptolide in rats after oral administration of triptolide (1 mg/kg, *n* = 6, mean ± S.D.) with or without puerarin pretreatment (100 mg/kg/day for seven days).

Parameter	Triptolide	Triptolide + puerarin
*T*_max_ (h)	0.51 ± 0.06	0.27 ± 0.2*
*C*_max_ (ng/mL)	187.25 ± 15.36	219.67 ± 21.52*
*t*_1/2_ (h)	1.03 ± 0.12	1.18 ± 0.18*
AUC_(0–_*_t_*_)_ (ng·h/mL)	201.55 ± 21.08	261.38 ± 31.27*
CL (L/h/kg)	4.92 ± 0.35	3.75 ± 0.19*

*p < 0.05 indicates significant differences from the control.

When the rats were pretreated with puerarin, the *C*_max_ and AUC_(0–_*_t_*_)_ of triptolide were increased significantly compared with the control (*p* < 0.05), and the *t*_1/2_, oral clearance and *T*_max_ of triptolide were significantly decreased than that of the control (*p* < 0.05). These results indicated that puerarin could significantly increase the plasma concentration of triptolide when they are co-administered.

### Effects of puerarin on the bidirectional transport of triptolide across Caco-2 cells

**Figure 3.**
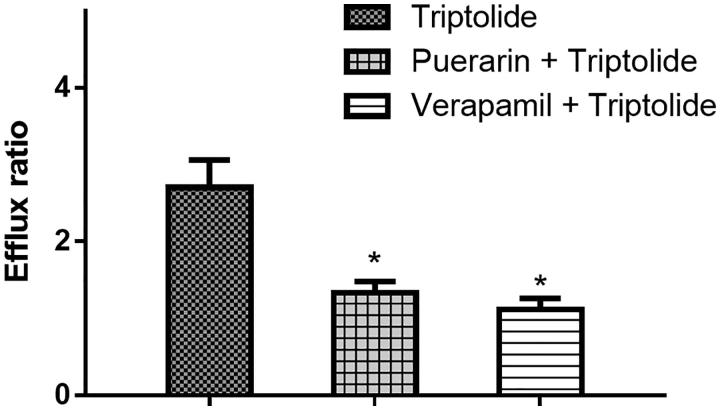
Effects of puerarin or verapamil on the efflux ratio of triptolide in the Caco-2 cell monolayer model. Each point represents the average ± S.D. of three determinations. *p < 0.05 indicates significant differences from the triptolide group.

To investigate the effects of puerarin on the transport of triptolide, the Caco-2 cell *in vitro* model was utilized. To validate the efflux activity of *P-gp*, a typical *P-gp* substrate digoxin was used, and the results indicated that the efflux ratio of digoxin was 10.28, which was abrogated in the presence of a typical *P-gp* inhibitor verapamil. The results indicated that the efflux activity of *P-gp* was qualified for the experiment. Then the transport of 2 μM of triptolide across Caco-2 cell monolayers was investigated in this study. As shown in Figure 3, the *P*_appAB_ and *P*_appBA_ were 2.15 ± 0.36 × 10^−7^ and 5.81 ± 0.46 × 10^−7^ cm/s, respectively. The *P*_appBA_ was much higher than the *P*_appAB_, and the efflux ratio of was 2.70, which indicated that efflux transporters might be involved in the transport of triptolide. Then, the transport studies were performed in the presence of puerarin (50 μM). In the presence of 50 μM of puerarin, the *P*_app_ values from the AP side to the BL side increased (3.29 ± 0.25 × 10^−7^ cm/s), whereas those from the BL side to the AP side decreased (4.37 ± 0.36 × 10^−7^ cm/s). The efflux ratio decreased from 2.70 to 1.33, and the efflux of triptolide was completely inhibited. In the presence of verapamil (20 μM, a typical *P-gp* inhibitor), the efflux ratio decreased from 2.70 to 1.12, and the efflux of triptolide was completely inhibited. These results indicated that puerarin could increase the absorption of triptolide through inhibiting the activity of *P-gp*.

### Effects of puerarin on the metabolic stability of triptolide in rat liver microsomes

The effects of puerarin on the metabolic stability of triptolide were investigated using rat liver microsomes. The metabolic half-life of triptolide was 35.7 ± 5.6 min, and the intrinsic clearance rate of triptolide was 38.8 ± 4.7 μL/min/mg protein, while in the presence of puerarin, the metabolic half-life (42.1 ± 7.3 min) was prolonged (*p* < 0.05), and the intrinsic clearance rate (32.9 ± 6.5 μL/min/mg protein) was decreased, and the difference was significant (*p* < 0.05). These results indicated that puerarin could slow down the metabolism of triptolide in rat liver microsomes and decrease the intrinsic clearance rate in rat liver.

## Discussion

Most studies investigate the effects of other herbs or drugs on the pharmacokinetics of puerarin. However, this is the first study to investigate the effects of puerarin on the pharmacokinetics of triptolide in rats and clarifies the main mechanism. The results indicated that puerarin could increase the absorption of triptolide as the *C*_max_ and AUC_(0–_*_t_*_)_ increased significantly with the pretreatment of puerarin. The *t*_1/2_ and oral clearance of triptolide decreased significantly, which suggested that the metabolism of triptolide might be inhibited. Previous studies have reported that puerarin could inhibit the activity of CYP3A4 and *P-gp* (Guo et al. 2014; Kim et al. 2014), and therefore, we speculated that puerarin might increase the absorption of triptolide by inhibiting *P-gp* mediated drug efflux or inhibiting CYP3A4 mediated metabolism.

To investigate the effects of puerarin on the absorption of triptolide and clarify its potential mechanism, the Caco-2 cell monolayer model was used. The results suggested that the efflux of triptolide was much higher than its influx, and puerarin could increase its absorption through inhibiting the activity of *P-gp*. The rat liver microsomes were also used to investigate the effects of puerarin on the metabolic stability of triptolide *in vitro*. These results revealed that puerarin could slow down the metabolism of triptolide in rat liver microsomes and decrease the intrinsic clearance rate in rat liver. As puerarin could inhibit the activity of CYP3A4, we inferred that puerarin could decrease the intrinsic clearance rate through inhibiting the activity of CYP3A4.

In conclusion, puerarin significantly changes pharmacokinetic profiles of triptolide in rats, and these results might be exerted though inhibiting the activity of *P-gp* or CYP3A4. Clinically, concomitant puerarin and triptolide treatment would allow dose reduction and still achieve comparable exposure of triptolide.
